# Childhood dental caries experience in northern Spain: a cross-sectional study

**DOI:** 10.1007/s40368-022-00762-2

**Published:** 2022-10-20

**Authors:** Jon Fernández-Bonet, Xabier Marichalar-Mendia, Aitana Lertxundi-Manterola

**Affiliations:** 1 Department of Nursing I, University of the Basque Country (UPV/EHU), Barrio Sarriena S/N, 48940 Leioa, Bizkaia Spain; 2 Department of Preventive Medicine and Public Health, University of the Basque Country (UPV/EHU), Barrio Sarriena S/N, 48940 Leioa, Bizkaia Spain

**Keywords:** Dental health, Dental health education, Dental services research, Caries

## Abstract

**Purpose:**

This study aimed to describe the caries experience in primary and permanent dentition of schoolchildren from a sample taken in public schools in Bilbao, to identify the most vulnerable child population, and compare them with the findings obtained by the Children’s Dental Care Programme in the region of the Basque Country (Spain).

**Methods:**

A cross-sectional study was conducted using a representative sample (*n* = 1682) of children from 5-year-old early childhood education classes and the first- and second-year elementary classes in public schools in Bilbao. The dependent variable was the caries experience determined through oral examinations carried out by a single dentist. On the other hand, independent variables were measured through questionnaires completed by families, with help from teachers as appropriate. In the statistical analysis, Mann–Whitney and Kruskal–Wallis nonparametric tests, as well as two logistic regressions, were performed, and the significance level was set at α = 0.05 for decision making.

**Results:**

The mean (SD) values of dft, DMFT, DMFS and DMFT of first permanent molars scores were 1.25 ± 2.20, 0.16 ± 0.61, 0.20 ± 0.90 and 0.15 ± 0.57, respectively. Compared to the findings in the most recent PADI report, the schoolchildren in our sample had slightly greater experience of dental caries in primary dentition and much greater experience in permanent dentition.

**Conclusion:**

The dft index of the primary dentition for the current sample is 1.25, while the DMFT index for the permanent dentition is 0.16. Among pupils in early childhood and elementary education in public schools in Bilbao, children from families with low socioeconomic status and educational attainment are most vulnerable to developing caries.

## Introduction

Dental caries is a chronic and multifactorial disease of the teeth, characterized by the progressive disintegration of calcified tissue. In children, it is one of the most prevalent diseases worldwide (Kassebaum et al. 2017), affects their oral health-related quality of life (OHRQoL) (Chaffee et al. 2017; Kramer et al. 2013) and is associated with an impact on their psychological well-being as well as difficulty chewing/eating and sleeping (Souza et al. 2018). Many children also develop other health problems associated with this disease, such as infections and pain (BaniHani et al. 2018). It results from microbiome dysbiosis with the involvement of multiple cariogenic species, including *Streptococcus mutans*, *Lactobacillus* species, *Scardovia wiggsiae*, and several *Actinomyces* species that have the cariogenic traits of acid production and tolerance (Zhan 2018). Caries experience is influenced by individual factors, such as the microbial composition of biofilms, amount of sucrose and refined carbohydrate in the diet, saliva flow, tooth morphology, time, fluoride in drinking water and toothpaste, and immunity to *Streptococcus mutans*, but also by social, environmental and cultural characteristics. Specifically, individuals of low socioeconomic status have poorer OHRQoL, regardless of the country’s economic classification, socioeconomic status indicator used and age group (Knorst et al. 2021).

The oral health of the school population of the autonomous region of the Basque Country (Spain) was first analyzed in 1988, finding a high prevalence of caries among children. As a result, two measures were adopted: the gradual fluoridation of waters and the implementation of the Children’s Dental Care Programme (PADI from the Spanish *Programa de Asistencia Dental Infantil*) (Department of Health Basque Government 2019). The PADI covers dental care for children aged 7–15 years, offering a free annual check-up in one of a list of public and associated private dental clinics, as well as restorative treatment of permanent dentition if necessary. Notably, in 2020, 37% of children eligible to go to a PADI check-up did not use this free dental service (Community Dental Service 2021). Studies carried out by the PADI every decade, however, have shown that the prevalence of dental caries has decreased in the Basque child population since 1988 (Department of Health Basque Government 1990, 1998, 2010). Indeed, in the 2018 report, the value of the Decayed, Missing and Filled Teeth (DMFT) index, a very widely used score for determining and monitoring the permanent dentition status in a population, was very low (0.04) among 7-year-old Basque schoolchildren (Department of Health Basque Government 2019).

Nonetheless, there is a lack of information on children who do not attend PADI check-ups. Some may be receiving adequate care through private providers, but we suspect that most are not attending any dental check-ups and hence represent a highly vulnerable population. The PADI reports also do not present the results by educational attainment of the family. Therefore, the caries rates provided by these studies may be biased and not reflect the complete picture because they do not include children who do not take advantage of this program.

This study aimed to describe dental caries experience in primary and permanent dentition of schoolchildren in a sample taken in public schools in the city of Bilbao, stratifying by sociodemographic characteristics seeking to identify vulnerable groups, and compare the findings with those obtained in studies of the PADI across the Basque Country. We hoped that the data gathered might help identify child populations in Bilbao’s public schools that may be more vulnerable to caries.

## Materials and methods

This is a descriptive cross-sectional study, which collected information on the oral health of school pupils through oral examinations. Sociodemographic variables were collected by distributing questionnaires to families, teachers offering to help as appropriate. The study was carried out in public schools in Bilbao, where the doctors and nurses of the City Council routinely carry out health promotion activities. One of these activities is to teach elementary school pupils the importance of proper brushing after each meal, as well as to how to brush their teeth. Conducting these workshops and speaking to the teachers, the medical staff of the municipality perceived a need to quantify the dental caries experience of these children.

The study population consisted of pupils from public schools in Bilbao, the most populous city in the Basque Country with a population of over 342 thousand in 2016 (Martín et al. 2018). The PADI studies used for comparisons were conducted in both public, maintained and private schools and across the Basque Country, that is, the entire region, which has a population of 2.16 million people (Department of Health Basque Government 1990, 1998, 2010, 2019).

In this study, the caries experience was evaluated in children from 5 to 7 years old. The sample was taken from the oldest pre-school classes (early childhood education for 5-year-olds) and first- and second-year classes in elementary education (for 6- and 7-year-olds). Schools were stratified by district (8 in total), and by simple random sampling, at least two schools were chosen per district. Overall, 21 public schools were included in the study (55.26% of all public schools in Bilbao).

Before the oral examination, the family of each schoolchild received an information sheet explaining the project, an informed consent form and a questionnaire consisting of a series of questions about their sociodemographic characteristics and the oral health of their children. If the families had poor literacy skills, the classroom teacher was asked to offer to help fill in the questionnaires with them.

To maintain the anonymity of participants, each child was assigned a unique identity number, which was written on the outside of an envelope, the consent form and the questionnaire, ensuring that all the data were de-identified. Informed consent was obtained from all participating families before the dentist performed the oral examinations. If the children did not bring back the informed consent form signed within 15 days, another form was given to them, in case the previous one had been lost. The study was approved by the ethics committee of the University of the Basque Country (M10_2016_157).

A variable describing occupation-based socioeconomic status was constructed from information on the occupation of the parents or guardians collected in the questionnaires. Specifically, the parents or guardians were assigned to one of three categories based on their current or most recent occupation: (1) non-manual; (2) intermediate, or (3) manual, which correspond to a simplified version of the three-class models of the European Socio-economic Classification (Rose and Harrison 2007) and UK National Statistics Socio-economic Classification rebased on the Standard Occupational Classification 2020 (UK Government 2020). In brief, the “non-manual” category was used for managers in the public administration or companies, professions associated with first-, second- and third-cycle university degrees, higher grade technical and supervisory positions, artists and sportsmen and women; and the “intermediate” category for administrative and clerical employees supporting administrative and financial management, personal and security services workers, self-employed workers not included in the “non-manual” class, and supervisors of manual workers; while routine occupations and skilled, semi-skilled and non-skilled manual workers, as well as those who had never worked, were classified in the “manual” category. On the other hand, parents or guardians were asked about their highest level of education. The responses were stratified into the following categories: (1) elementary; (2) compulsory secondary; (3) higher secondary or vocational training; and (4) university. Children were assigned the highest socioeconomic status and level of education among those of their mother and/or father or legal guardian. If the parents/guardians did not provide information concerning their occupation or educational attainment but did give informed consent for oral exploration, children were classified into a fourth group: “not reported”.

Oral examinations were performed over a year, between May 2016 and May 2017. A single dentist with a master's degree in public health and 4 years of experience working as a clinician performed all the examinations for the study. The dental examinations were performed in the medical offices of each of the schools with a flat mirror no. 5, an exploration probe and a periodontal probe for each pupil, following World Health Organization recommendations as in the PADI studies (World Health Organization 2013). In line with these recommendations, caries was recorded as present when a lesion in a pit or fissure, or on a smooth tooth surface, had an unmistakable cavity, undermined enamel, or a detectably softened floor or wall, and these diagnostic criteria were submitted to the Bilbao City Council's Head of Health and Consumer Affairs before the study. No bitewing or other X-ray examinations were taken. The teeth were dried using cotton wool and while most explorations were carried out with natural light, an artificial light source (100-W white light lamp) was used when required (to ensure sufficient light).

The dentist went to all the schools several times on different days, to try to examine all the children whose parents had given written informed consent. Further, he repeated the first 50 examinations, as well as 10% of the entire sample of examinations, for diagnostic reliability testing.

The caries experience was calculated like in the PADI studies used for comparisons. First, for each of the children, DMFT is the sum of the number of Decayed, Missing due to caries, and Filled Teeth in their permanent dentition and the mean for the population, the DMFT index, is the sum of individual DMFT values divided by the total number of children assessed (Varenne 2021). Second, DMFS is the sum of the number of Decayed, Missing due to caries, and Filled Surfaces in the permanent dentition, and similarly, the DMFS index is the sum of all individual DMFS values divided by the total number of children in the sample. Third, the DMFT index of the first permanent molars is calculated like the DMFT index but taking into consideration just the first molars instead of all the permanent teeth. Lastly, the dft is the sum of the number of Decayed and Filled Teeth in the primary dentition and the dft index is the sum of individual dft values divided by the total number of individuals in the sample.

The oral health of the sample was described by the aforementioned caries experience (DMFT, DMFS, DMFT of first permanent molars and dft) using the means (the indices) and corresponding standard deviations. The scores of the permanent dentition were only calculated in the children ≥ 6 years of age, while the dft index was calculated for the entire sample. With data on DMFT, DMFS and dft available from the most recent PADI study (private communication), we assessed differences in the mean values of these indices between the cohorts using Student’s *t* tests. For our sample, values of the four caries indices were then compared by sex, family educational attainment and socioeconomic status. For this, Mann–Whitney and Kruskal–Wallis nonparametric tests were performed, and the significance level was set at α = 0.05.

Two logistic regression models were also constructed to identify explanatory variables for dental caries in our sample, one for the primary dentition and the other for the permanent dentition. In these models, we excluded children who lacked data on family socioeconomic status or educational attainment. After excluding these cases, the sample was composed of 1265 children for the primary dentition and 931 for the permanent dentition.

This regression analysis was performed taking the dft and DMFT index values as a reference. In the dependent variables of the regressions, the children with dft/DMFT ≥ 1 were assigned to the "disease group", while those with dft/DMFT = 0 were assigned to the “no-disease group”. Before running the regression, collinearity diagnostics were applied, and VIF scores indicated that there was no multicollinearity.

## Results

**Fig. 1:**
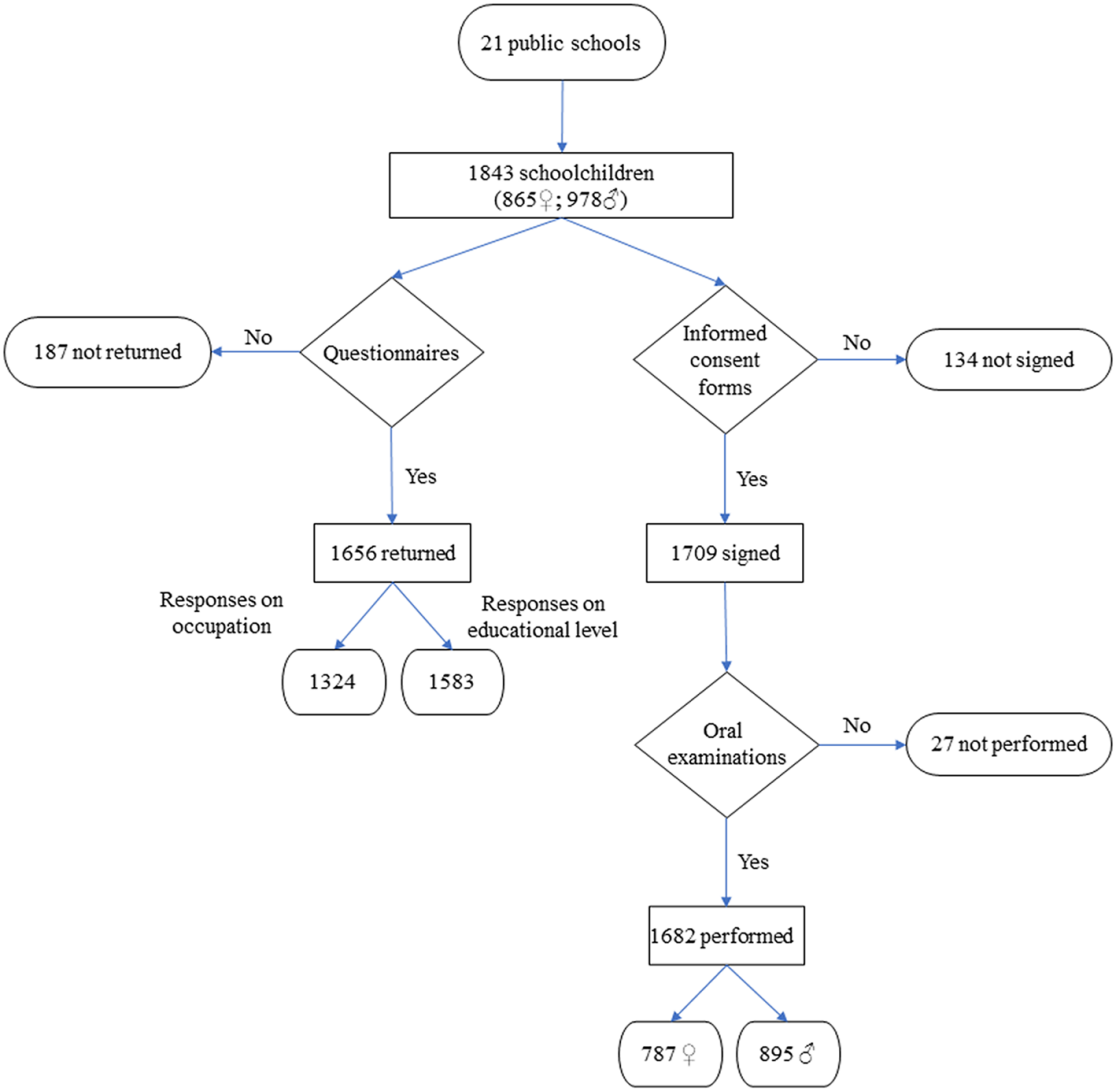
Flowchart explaining the process of obtaining the sample

A total of 1843 children enrolled in the schools selected for this project were eligible to participate. Finally, 1682 children took part in the project, with a mean age of 6.77 ± 0.97 years at the start of the study. The participation rates in each year group were as follows: 94.67% in the pre-school classes (5-year-olds), and 93.88% and 90.2% in the first- and second-year elementary education classes respectively (6- and 7-year-olds). Some oral examinations were not performed despite parental consent because the children were not in class at the time or moved to a new school (Fig. 1 and Table 1). Rates of response to the questionnaires and provision of written informed consent to the oral examination were influenced by the sociodemographic characteristics of the families; this may represent a selection bias (Appendixes 1–4). In the diagnostic reliability testing, good intra-rater agreement was observed (95% concordance).

**Table 1:** Description of the total sample (*n* = 1843)

	*n* (%)
*Sex*	
Boys	978 (53.1%)
Girls	865 (46.9%)
*School class*	
Last year of preschool (for 5-year-olds)	544 (29.5%)
1st year of elementary (for 6-year-olds)	605 (32.8%)
2nd year of elementary (for 7-year-olds)	694 (37.7%)
*Family member unemployed*^a^	
Yes	812 (44.1%)
No	691 (37.5%)
Not recorded	340 (18.4%)
*Educational attainment*	
University	453 (24.6%)
Higher secondary/vocational training	629 (34.1%)
Compulsory secondary	215 (11.7%)
Elementary	286 (15.5%)
Not recorded	260 (14.1%)
*Socioeconomic status*	
Non-manual	277 (15%)
Intermediate	179 (9.7%)
Manual	868 (47.1%)
Not recorded	519 (28.2%)
*Written informed consent*	
Given	1709 (92.7%)
Not given	134 (7.3%)
*Oral examinations*	
Performed	1682 (90.3%)
Not performed	181 (9.7%)

^a^At least one member of the household unemployed

Dental caries data are reported overall and by child sex, family educational attainment and socioeconomic status (Table 2). The mean age of the children for whom the dft score was calculated was 6.77 ± 0.97 years, while the mean age was 7.20 ± 0.73 years among the children for whom we assessed permanent dentition scores. Regarding primary dentition, the dft index of the current sample was 1.25, comparable to the value of 1.15 cited in the most recent PADI report (*p* > 0.05). On the other hand, both DMFT and DMFS values were strikingly higher in this study: 0.16 and 0.20 respectively, compared to a value of 0.04 for both indices in the PADI report; these differences were statistically significant (*p* < 0.001).

**Table 2:** Dental caries experience overall and stratified by sociodemographic characteristics (standard deviations in parentheses)

	*n*	Mean dft^a^	*n*	Mean DMFT^b^	Mean DMFS^c^	Mean DMFT 1st Molar^d^
All combined	1682	1.25 **(**2.20)	1249	0.16 (0.61)	0.20 **(**0.90)	0.15 **(**0.58)
*Sex*						
Boys	895	1.35 **(**2.34)	671	0.13 (0.52)	0.15**(**0.63)	0.13 **(**0.50)
Girls	787	1.14**(**2.03)	578	0.19 (0.70)	0.25 **(**1.13)	0.18 **(**0.65)
*Educational level*						
University	445	0.67 **(**1.60)^**^	315	0.04 **(**0.24)^**^	0.05 **(**0.31)^**^	0.03 **(**0.23)^**^
Higher secondary/vocational training	615	1.03 **(**1.98)	461	0.10 **(**0.42)	0.10 **(**0.47)	0.10 **(**0.41)
Compulsory secondary	205	1.36 **(**2.15)	152	0.15 **(**0.58)	0.19 **(**0.82)	0.15 **(**0.58)
Elementary	270	2.11 **(**2.68)	174	0.45 **(**1.07)	0.61 **(**1.74)	0.42 **(**0.96)
Not recorded	147	2.19 **(**2.90)	147	0.26 (0.80)	0.31 **(**1.19)	0.24 **(**0.77)
*Socioeconomic status*						
Non-manual	274	0.59 **(**1.71)^**^	187	0.05 **(**0.28)^*^	0.05 (0.30)^*^	0.04 **(**0.25)^*^
Intermediate	178	0.58 **(**1.27)	132	0.06 **(**0.30)	0.06 **(**0.30)	0.06 **(**0.30)
Manual	833	1.36 **(**2.20)	632	0.18 **(**0.65)	0.22 **(**1.02)	0.17 (0.61)
Not recorded	397	1.78 **(**2.62)	298	0.23 **(**0.74)	0.29 **(**1.04)	0.21 **(**0.71)

^a^Decayed or Filled teeth score (all children in the sample)

^b^Decayed, Missing or Filled teeth score (≥ 6-year-olds)

^c^Decayed, Missing or Filled Surfaces score (≥ 6-year-olds)

^d^Decayed, Missing or Filled score of the first permanent molars (≥ 6-year-olds)

*p < 0.01; Kruskal–Wallis *H*-Test. **p < 0.001; Kruskal–Wallis *H*-test

In our sample, dental caries experience was greater in boys in the case of primary dentition, while it was higher in girls in the case of permanent dentition. Children from families with lower socioeconomic status and educational attainment had greater dental caries experience in both the primary and permanent dentition. Children whose parents gave written informed consent but who lacked data on their family’s sociodemographic characteristics also had high caries experience.

To strengthen caries prevention, it is crucial to identify explanatory variables for the development of caries in both primary and permanent dentition. The results of the binary logistic regressions for our sample suggest that family educational attainment and socioeconomic status strongly influence the occurrence of the disease in children’s primary dentition, while child age and sex together with family educational attainment are key factors in the case of their permanent dentition (Table 3). Regarding socioeconomic status, pairwise comparisons of dft index values show statistically significant differences between the manual group and the rest with an exception: the manual group versus the group whose socioeconomic status was not recorded (Appendix 5).

**Table 3:** Estimation of the odds ratio related to dental caries experience in the primary and permanent dentition

Sociodemographic variables		Primary dentition (*n* = 1265)		Permanent dentition(*n* = 931)	
		OR	95% CI	OR	95% CI
Age	Numerical variable	1.10	(0.97–1.25)	2.81	(1.68–4.72)
Sex	Girls	0.86	(0.67–1.09)	2.40	(1.08–5.33)
	Boys	Ref.			
Family member unemployed	Yes	1.24	(0.95–1.62)	1.10	(0.45–2.72)
	No	Ref.			
Socioeconomic status	Manual	1.96	(1.30–2.96)	1.28	(0.21–7.80)
	Intermediate	1.19	(0.74–1.92)	1.25	(0.16–9.78)
	Non-manual	Ref.		Ref.	
Educational attainment	Elementary	2.57	(1.62–4.07)	8.64	(1.62–46.10)
	Compulsory secondary	1.43	(0.92–2.24)	3.22	(0.56–18.57)
	Higher secondary/vocational training	0.96	(0.68–1.35)	1.52	(0.31–7.51)
	University studies	Ref.		Ref.	

**Fig. 2:**
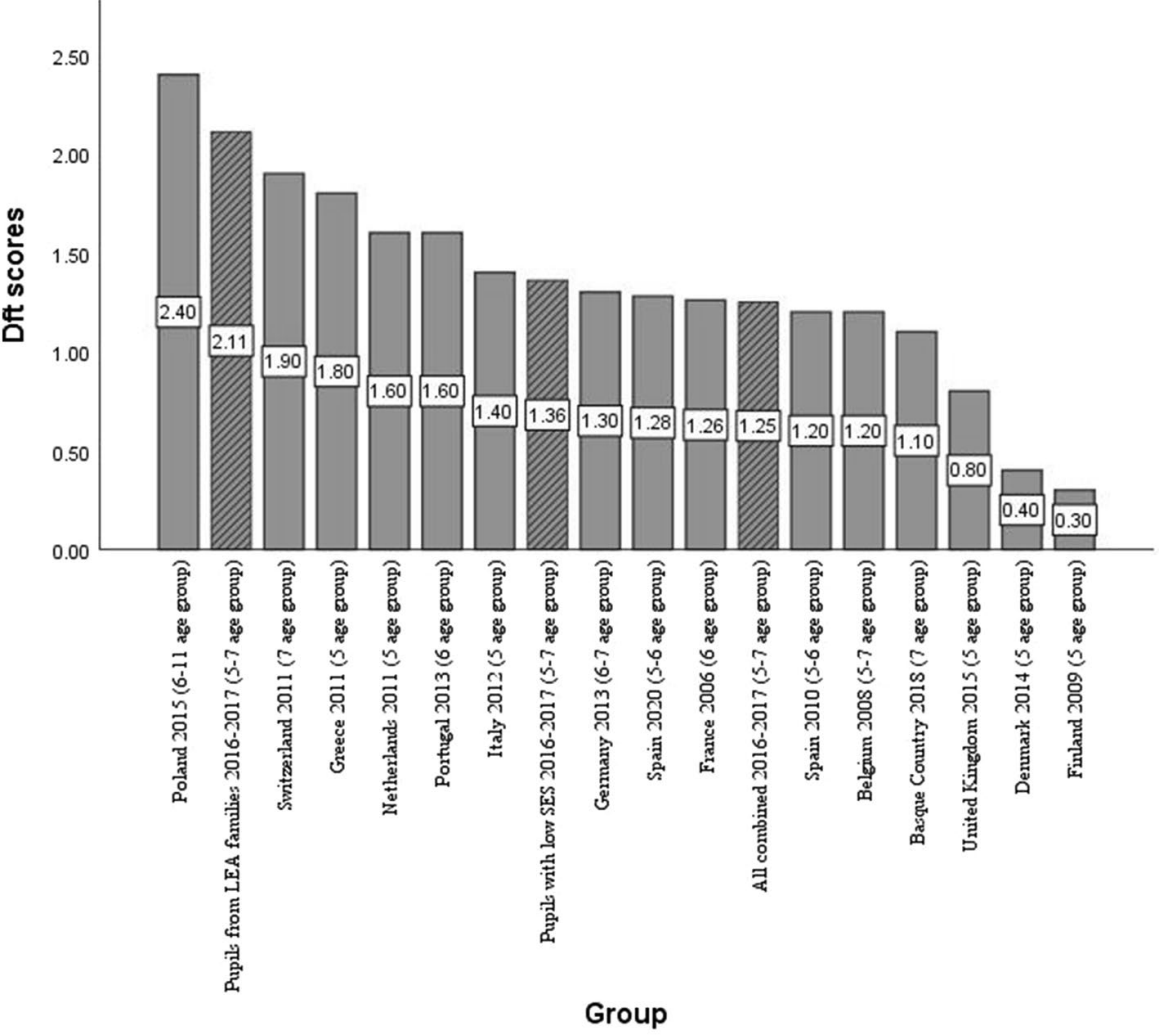
Comparison of dft index in groups in our study (hashed bars) with values in children of similar ages found in other European countries (solid bars) (Bravo-Pérez et al. 2016; Bravo-Pérez et al. 2020, Department of Health Basque Government 2019, Malmö University 2021). Abbreviations: LEA families: families with low educational attainment, that is, among whom elementary education was the highest level of education completed by the parents or guardians; SES: socioeconomic status based on the occupation of their parents or guardians

**Fig. 3:**
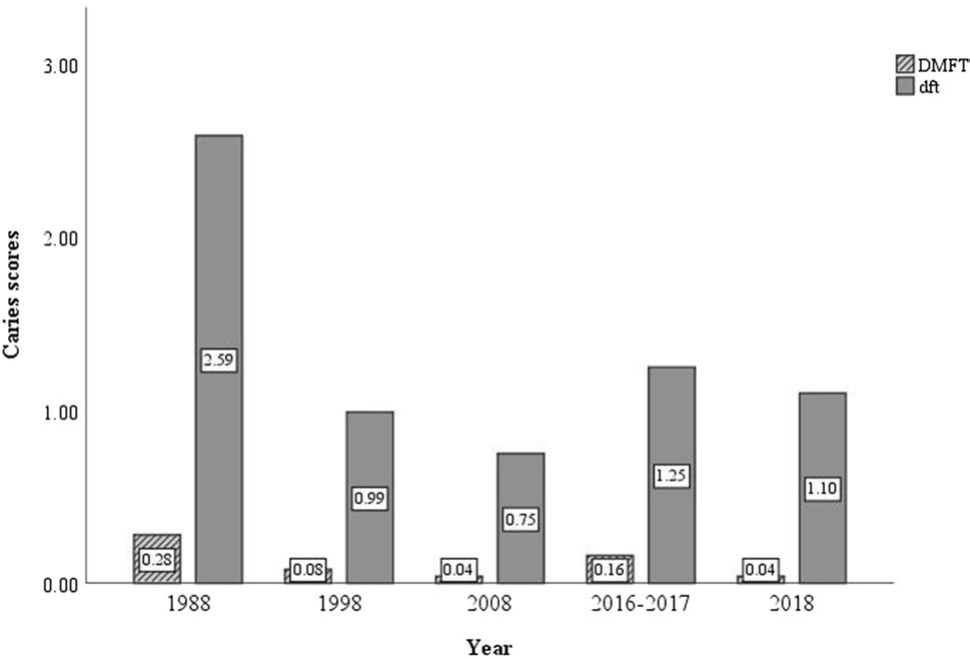
Comparison of caries experience observed in our study (2016–2017) with that reported in PADI studies (1988, 1998, 2008 and 2018) (Department of Health Basque Government 1990, 1998, 2010, 2019)

Finally, to provide context for interpreting our findings, Fig. 2 lists the dft index values from our study together with those from studies in other European countries (Bravo-Pérez et al. 2016; Bravo-Pérez et al. 2020; Department of Health Basque Government 2019, Malmö University 2021), while Fig. 3 shows the caries experience in the Basque Country over time (Department of Health Basque Government 1990, 1998, 2010).

## Discussion

This study aimed to describe the dental caries experience in primary and permanent dentition of schoolchildren from a sample taken in public schools in Bilbao, stratifying by sociodemographic characteristics to identify the most vulnerable child populations, and also to compare these findings with those of the region’s Children’s Dental Care Programme, in particular, the most recent PADI study (Department of Health Basque Government 2019). The caries index values found in the current study are relatively similar to those from the most recent PADI study for primary dentition but much higher for permanent dentition. In our sample, family socioeconomic status and educational attainment seem to play a fundamental role in the occurrence of caries in primary dentition, while child age and sex, as well as family educational attainment, play a crucial role in permanent dentition.

Concerning primary dentition, the higher scores in our sample could be explained by a large percentage of participants having the lowest socioeconomic status. In a recent study, lack of supervision of health behaviors in 6- to 7-year-olds was positively associated with dft and unhealthy oral health behaviors (Matsuyama et al. 2020). It seems reasonable to suppose that in groups with lower socioeconomic status there may be both less family knowledge and less involvement in caries prevention. Notably, the PADI report cited a dft of 1.3 for the manual socioeconomic group, similar to the value of 1.36 in our study.

Regarding permanent dentition, the dental caries experience observed in our research might be an overestimate due to the aforementioned selection bias. On the other hand, the PADI report scores may be an underestimate, as children from families with low socioeconomic status and educational attainment are more likely to be absent from school and if the dentists do not attend the same school several times, as was done in this study, they often do not take part in research studies.

The findings stratified by sex merit discussion. In primary dentition, the caries experience is higher in boys, while in permanent dentition, it is higher in girls. The explanation for why girls in this study have higher caries experience in the permanent dentition at such a young age may be two-fold: a slightly earlier eruption of the permanent dentition than in boys, this allowing more time to develop disease (Plasencia et al. 2021; Valdez et al. 2014; Virtanen et al. 1994) and their potentially less privileged position within certain families with low socioeconomic status and educational attainment (Aisa et al. 2016; Llorent-Verma and Torres-León 2012).

In relation to sociodemographic characteristics, the latest PADI report showed that 12–15% of children account for 75–80% of all caries observed in permanent dentition at 12–14 years of age, establishing a vulnerable population. In particular, it pointed out that children in the non-manual socioeconomic group had half as much experience of caries as those in the manual group. In our study, the DMFT index of the manual socioeconomic group is almost four times higher than that of the non-manual socioeconomic group. Considering that our study was carried out with a younger sample (mean age 7.20 years), the results are even more alarming. Logistic regression analysis suggests that age plays a key role in the development of caries in permanent dentition. That is, the older the children, the more time for the disease to occur, and hence, the real differences at 12 years of age between pupils with different sociodemographic characteristics can be expected to be even more marked than those found by the PADI.

It has already been shown that families with low educational attainment make less use of public dental care services in Spain (Jiménez et al. 2004; Pinilla et al. 2015; Tapias et al. 2011) and that the higher the level of education of the mother, the lower the levels of caries in the children (Smyth and Caamaño 2005). The role of the mother can therefore be considered essential in the prevention of early childhood caries (Paglia 2019). This is explained by cultural norms and values, which influence and reinforce health-related thoughts and behaviors (Abel 2008). Indeed, less than twice-daily tooth brushing and difficulties performing the procedure in the first preschool years may be significant determinants of caries prevalence at 5 years of age (Boustedt et al. 2020).

Given all the above, it is clearly necessary to facilitate the use of PADI by the most vulnerable groups in the city of Bilbao, promoting health education, hygiene and good oral care habits. There is also a need to differentiate between the use of a program by the population and coverage, which expresses the extent of interaction between the service and the target population (Tanahashi 1978). Consequently, it is essential to identify barriers and facilitators that hinder or favor the achievement of effective coverage in each vulnerable group (Frenz and Vega 2021; Tanahashi 1978). As the Basque Government has invested considerable resources in the PADI, and to a lesser extent, the fluoridation of the water, it is important from a cost-effectiveness point of view to address the question of whether these resources could be better spent focusing on health promotion among schoolchildren with certain sociodemographic characteristics, in particular, those with parents with low educational attainment and socioeconomic status.

The reason for the marked difference in caries experience in permanent dentition between our study and the PADI research remains unknown. There is a need for further studies including children from private and maintained as well as public schools in Bilbao, in particular, to explore whether – as we suspect might be the case—the caries experience would be, on average, more similar to those of the latest PADI study. It would also be interesting to follow up the children in this sample to 12–14 years of age to carry out a prospective study and explore any differences with the findings of the PADI report at this older age.

Among the strengths of this study, we should highlight the successful inclusion of children from families with low socioeconomic status and educational attainment in the sample, a group that is often absent from oral health research, as it tends to be difficult to obtain written informed consent from their families and enroll them in studies. Moreover, we believe that many of these children do not regularly attend the free check-ups offered (through the PADI). Further, a single dentist carried out all the oral exams, following the same diagnostic criteria. On the other hand, as weaknesses of the current study, it must be underlined that it was carried out only in public schools, not in private or maintained schools, which implies a selection bias. In addition, the data were not gathered using the International Caries Detection and Assessment System, which allows both the detection of carious lesions at an early stage and coding of disease progression to advanced stages in a standardized way. That is, our data do not capture the stage of the caries process, though the scores used have been widely employed for assessing caries prevalence, and in our case, facilitate comparisons with the findings of previous studies in our region. Finally, the dental examiner who carried out the examinations did not receive specific training for this study and his assessments were not calibrated against those of a gold standard examiner (inter-rater reliability), but he was experienced in caries assessment and achieved good diagnostic agreement (intra-rater reliability). We must therefore exercise caution in taking these findings as a reference for the oral health of all schoolchildren in Bilbao or the region of the Basque Country.

## Conclusion

Considering the limitations of the present study the following conclusions can be made:

1. The dft index of the primary dentition for the current sample is 1.25, while the DMFT index for the permanent dentition is 0.16.
2. Among pupils in early childhood and elementary education in public schools in Bilbao, children from families with low socioeconomic status and educational attainment are most vulnerable to developing caries.
3. Compared to the findings in the most recent PADI report, the schoolchildren in our sample had slightly greater experience of dental caries in primary dentition and much greater experience in permanent dentition.

## References

[CR1] Abel T (2008). Cultural capital and social inequality in health. J Epidemiol Community Health.

[CR2] Aisa R, Larramona G, Pueyo F (2016). Discrimination and self-reported health for the Spanish Roma. BMC Public Health.

[CR3] BaniHani A, Deery C, Toumba J (2018). The impact of dental caries and its treatment by conventional or biological approaches on the oral health-related quality of life of children and carers. Int J Paediatr Dent.

[CR4] Boustedt K, Dahlgren J, Twetman S (2020). Tooth brushing habits and prevalence of early childhood caries: a prospective cohort study. Eur Arch Paediatr Dent.

[CR5] Bravo-Pérez M, Almerich-Silla JM, Ausina-Márquez V et al. Encuesta de salud oral en España 2015. RCOE. 2016;21(Supl. 1):8–48. https://core.ac.uk/download/pdf/78633215.pdf Accessed Nov 29, 2021.

[CR6] Bravo-Pérez M, Almerich-Silla JM, Canorea-Díaz E et al. Encuesta de salud oral en España 2020. RCOE, 2020;25(4):7–35. https://sespo.es/wp-content/uploads/Encuesta-de-Salud-Oral-en-Espa%C3%B1a-2020.pdf Accessed Nov 29, 2021.

[CR7] Chaffee BW, Rodrigues PH, Kramer PF (2017). Oral health-related quality-of-life scores differ by socioeconomic status and caries experience. Commun Dent Oral Epidemiol.

[CR8] Community Dental Service. PADI - 2020. Annual report on the development of decree 118/90, on dental care for children in the autonomous community of the Basque Country. Vitoria-Gasteiz: Central Publications Service, Basque Government; 2021. https://www.euskadi.eus/contenidos/informacion/salud_padi/es_def/adjuntos/Informe_anual_PADI_2020-v2.pdf. Accessed 29 Nov 2021.

[CR9] Department of Health Basque Government (1990). 1st epidemiological study of children's oral health in the Basque Autonomous Community (1988).

[CR10] Department of Health Basque Government (1998). 2nd epidemiological study of children's oral health in the Basque Autonomous Community (1998).

[CR11] Department of Health Basque Government (2010). 3rd epidemiological study of children's oral health in the Basque Autonomous Community (2008).

[CR12] Department of Health Basque Government. 4th epidemiological study of children's oral health in the Basque Autonomous Community (2018). Vitoria-Gasteiz: Central Publications Service, Basque Government; 2019. https://www.euskadi.eus/contenidos/informacion/salud_padi/es_def/adjuntos/Salud-Dental-Inf-conclusiones.pdf. Accessed 29 Nov 2021.

[CR13] Frenz P, Vega J. Universal health coverage with equity: What we know, don't know and need to know. Background paper for the global symposium on health systems research; November 16–19, 2010; Montreux, CH. https://healthsystemsresearch.org/hsr2010/images/stories/9coverage_with_equity.pdf Accessed Nov 30, 2021.

[CR14] Jiménez R, Tapias-Ledesma MA, Gallardo-Pino C (2004). Influence of sociodemographic variables on use of dental services, oral health and oral hygiene among Spanish children. Int Dent J.

[CR15] Kassebaum NJ, Smith A, Bernabé E, et al. Global, regional, and national prevalence, incidence, and disability-adjusted life years for oral conditions for 195 countries, 1990–2015: a systematic analysis for the global burden of diseases, injuries, and risk factors. J Dent Res. 2017;96(4):380–387. 10.1177/0022034517693566 10.1177/0022034517693566 PMC5912207 28792274

[CR16] Knorst JK, Sfreddo CS, Zanatta FB (2021). Socioeconomic status and oral health-related quality of life: A systematic review and meta-analysis. Commun Dent Oral Epidemiol.

[CR17] Kramer PF, Feldens CA, Helena Ferreira S (2013). Exploring the impact of oral diseases and disorders on quality of life of preschool children. Commun Dent Oral Epidemiol.

[CR18] Llorent-Vermar V, Torres-León N (2012). Lifelong learning of Gypsy women in Spain. Procedia Soc Behav Sci.

[CR19] Malmö University. Oral Health Country/Area Profile Project. Geneva (Switzerland)/ Malmö (Sweden): The World Health Organization and Malmö University; 2021. https://capp.mau.se/country-areas/. Accessed Nov 29, 2021.

[CR20] Martín U, González-Rábago Y, Bacigalupe A et al. Bilbao's health in figures: a quantitative diagnosis. Bilbao: University of the Basque Country (UPV/EHU), Health and Consumption Area of the City Council of Bilbao; 2018. https://www.ehu.eus/documents/3638427/0/La+Salud+de+Bilbao+en+cifras.Un+diagn%C3%B3stico+cuantitativo.pdf/d9d5f4b0-97c5-60cd-866a-493e966d99cd?t=1575897137000. Accessed Nov 30, 2021.

[CR21] Matsuyama Y, Isumi A, Doi S et al. Poor parenting behaviours and dental caries experience in 6- to 7-year-old children. Commun Dent Oral Epidemiol. 2020;48(6):493–500. 10.1111/cdoe.12561 10.1111/cdoe.12561 PMC7689935 32750206

[CR22] Paglia L. Oral prevention starts with the mother. Eur J Paediatr Dent. 2019;20(3):173. 10.23804/ejpd.2019.20.03.01 10.23804/ejpd.2019.20.03.01 31489813

[CR23] Pinilla J, Negrín-Hernández MA, Abásolo, I. Time trends in socio-economic inequalities in the lack of access to dental services among children in Spain 1987–2011. Int J Equity Health. 2015;14(9) 10.1186/s12939-015-0132-8 10.1186/s12939-015-0132-8 PMC4316659 25636711

[CR24] Plasencia E, García-Izquierdo F, Puente-Rodríguez M. Edad de emergencia y secuencias polimórficas de la dentición permanente en una muestra de población de Asturias. RCOE. 2005:10(1):31–42. https://scielo.isciii.es/scielo.php?script=sci_arttext&pid=S1138-123X2005000100003 Accessed Nov 30, 2021.

[CR25] Rose D, Harrison E (2007). The European Socio-Economic Classification: A new social class schema for comparative European research. Eur Soc.

[CR26] Smyth E, Caamaño F (2005). Factors related to dental health in 12-year-old children: a cross-sectional study in pupils. Gac Sanit.

[CR27] Souza JGS, Souza SE, Noronha MdS et al. Impact of untreated dental caries on the daily activities of children. J Public Health Dent. 2018;78(3):197–202. 10.1111/jphd.12259 10.1111/jphd.12259 29193108

[CR28] Tanahashi T (1978). Health service coverage and its evaluation. Bull World Health Organ.

[CR29] Tapias-Ledesma MA, Carrasco-Garrido P, Esteban y Peña M et al. Use of dental care and prevalence of caries among immigrant and Spanish-born children. J Dent Child (Chic). 2011;78(1):36–42. 22041007

[CR30] UK Government. SOC 2020 Volume 3: the National Statistics Socio-economic Classification (NS-SEC rebased on the SOC 2020); 2020 https://www.ons.gov.uk/methodology/classificationsandstandards/standardoccupationalclassificationsoc/soc2020/soc2020volume3thenationalstatisticssocioeconomicclassificationnssecrebasedonthesoc2020 Accessed Jun 2, 2021.

[CR31] Valdez-Penagos RG, Sánchez-Acuña G, Romo-Pinales MR (2014). Edad media de la erupción dental en una población escolar analizada por dos métodos. Bol Med Hosp Infant Mex.

[CR32] Varenne B. Mean number of decayed, missing, and filled permanent teeth (mean DMFT) among the 12-year-old age group. https://www.who.int/data/gho/indicator-metadata-registry/imr-details/3812. Accessed Nov 30, 2021.

[CR33] Virtanen JI, Bloigu RS, Larmas MA. Timing of eruption of permanent teeth: Standard Finnish patient documents. Community Dent Oral Epidemiol. 1994;22(5PT1):286–288. 10.1111/j.1600-0528.1994.tb02052.x 10.1111/j.1600-0528.1994.tb02052.x 7813177

[CR34] World Health Organization. Oral health surveys: basic methods - 5th edition. Geneva: WHO; 2013. https://apps.who.int/iris/bitstream/handle/10665/97035/9789241548649_eng.pdf?sequence=1&isAllowed=y. Accessed Nov 30, 2021.

[CR35] Zhan L (2018). Rebalancing the Caries Microbiome Dysbiosis: Targeted Treatment and Sugar Alcohols. Adv Dent Res.

